# The Status of Dietary Energy and Nutrients Intakes among Chinese Elderly Aged 80 and Above: Data from the CACDNS 2015

**DOI:** 10.3390/nu13051622

**Published:** 2021-05-12

**Authors:** Fanglei Zhao, Li He, Liyun Zhao, Qiya Guo, Dongmei Yu, Lahong Ju, Hongyun Fang

**Affiliations:** National Institute for Nutrition and Health, Chinese Center for Disease Control and Prevention, No. 27 Nanwei Road, Xicheng District, Beijing 100050, China; zfl6869@163.com (F.Z.); heli@ninh.chinacdc.cn (L.H.); zhaoly@ninh.chinacdc.cn (L.Z.); guoqy@ninh.chinacdc.cn (Q.G.); yu_dongmei@126.com (D.Y.); julh@ninh.chinacdc.cn (L.J.)

**Keywords:** dietary, energy, nutrients, oldest-old, China

## Abstract

This study analyzed the status of dietary energy and nutrients intakes among the oldest-old in China. Data was obtained from the China Adult Chronic Disease and Nutrition Surveillance in 2015 (CACDNS 2015). We enrolled 1929 Chinese elderly people aged 80 and above who participated in both 3-day 24-h dietary recalls and household condiments weighing. The dietary intakes were calculated based on Chinese Food Composition Tables and assessed using Chinese Dietary Reference Intakes (DRIs). The dietary intakes of energy and most nutrients were all below the EAR or AI, except for fat, vitamin E, niacin, iron and sodium. As a result, daily dietary intakes of energy and most nutrients were inadequate in the oldest-old in China, especially vitamin A, vitamin B_1_, vitamin B_2_, folate and calcium, with the prevalence of deficiency more than 90%. Furthermore, the prevalence of inadequacy of vitamin C, zinc, selenium and magnesium was also high with the proportion below the EAR more than 60%. Approximately 30% of the subjects with dietary vitamin E intake did not reach AI, and more than 90% of subjects have reached AI in the intake of sodium, while more than 90% did not reach AI in potassium. The mean intakes of niacin and iron have reached EAR, but around 15% were still faced with the risk of deficiency. In addition, although the dietary energy intake was below EER, the energy contribution from fat in total population and all subgroups (region, age, gender, education level, material status, household income level groups) all exceeded the recommended proportion of 30% from the DRIs and close to or over 35%, is a significant concern. For the majority of nutrients, higher daily dietary intakes and lower prevalence of deficiencies were found in the oldest-old living in urban areas, aged 80–84 years, with high school and above education level, living with spouse and from high household income family. These findings indicates that the dietary intakes of energy and nutrients were inadequate, while the energy contribution from fat and dietary sodium intake were too high among the oldest-old in China. Most oldest-old were at high risk of nutritional deficiency, particularly for those who living in rural areas, with lower education level and from low household income.

## 1. Introduction

The world population is ageing rapidly due to advancements in nutrition, sanitation, health care, education and economic well-being. What is more, the number of oldest-old (those aged 80 and above) is dramatic increasing and even faster than the elderly aged 65 and above [1]. According to World Population Prospects 2019, the number of world’s population aged 80 years and above is projected to triple by 2050, from 143 million in 2019 to 426 million [1], which brings a huge challenge for health and social care system in all countries.

The basal metabolism, physiological function and physical activity declined with age. As a result, the elderly were more likely to suffer health problems including poor handgrip strength, anemia, hip fracture, malnutrition, fatigue, cardiovascular disease, cognitive and frailty, back and neck pain and osteoarthritis, chronic obstructive pulmonary disease, diabetes, depression and geriatric syndromes [2,3,4,5,6,7,8,9]. Furthermore, elderly people tend to suffer from multiple diseases that may require more medical care and daily assistance in the world.

Generally speaking, the people aged 60 and over are called old people, and the people aged 80 or more are called oldest-old in China. As we all know, with the increase of age, chewing ability, digestive function, appetite and physical activity decrease, resulting in the decrease of nutrients and food intakes. It is reported that the daily dietary energy and nutrients intakes among old people were all higher than the oldest-old [10,11,12,13]. What is more, according to DRIs, the EAR or AI of most nutrients in old people and oldest-old were the same, and the EER of energy, EAR of magnesium and niacin, and the AI of sodium were slightly lower in oldest-old than in old people. However, it is estimated that the prevalence of deficiency for nearly all nutrients was higher in the elderly aged 75 and over than the elderly aged 60 and over based on China National Nutrition and Health Surveillance (CNNHS) 2010–2012 [11]. The above results indicated that the deficiency of dietary nutrients was more serious in the oldest-old than in old people. When compared to old people in CNNHS 2010–2012 [10] and China Health and Nutrition Surevy (CHNS) 2015 [12], energy contribution from carbohydrate was lower, but energy contribution from fat was higher in oldest-old, which highlighted that energy contribution from macronutrients was more unreasonable in oldest-old. As for physical activity, the proportion of not going out was higher in the elderly aged 75 and over compared to old people, and sedentary time in leisure life was longer than old people, while the time of going out and doing housework were less than old people in CNNHS 2010–2012 [11]. In addition, in terms of nutritional status, the prevalence of anemia, malnutrition, hypertension and diabetes was higher in the elderly aged 75 and over compared to old people aged 60 and over [14]. In a word, the result showed that dietary nutrition in oldest-old was worse than old people, and more attention should be paid to oldest-old.

It is predicted that China is going to enter a deep aging society in 2022 [15]. There are 21.0 million oldest-old in China based on 2010 sixth national population census data [16]. The old people have about 1.61 chronic diseases and approximately 44.46% of them has two or more chronic diseases in China [17]. The rapid rise in the prevalence of chronic diseases has resulted in an increased burden of disability [18]. The Fourth Sample Survey on the Living Conditions of Elderly People in Urban and Rural China (2015) reported that China has 40.63 million partially and completely disabled elderly, accounting for 18.3% of total elderly, and 41.0% of those aged 80 and above needed care services [19]. This condition will lead to marked increase of public health resource consumption and medical health expenditure.

Balanced diet is the crucial way to prevent chronic diseases and improve life quality. Having an ideal diet was associated with lower mortality among the oldest-old [20]. However, few studies on dietary intakes of oldest-old were conducted in China. This study aimed to analyze the dietary intakes of energy and nutrients among the elderly aged 80 and above, and to assess deficiency risk in total population and subgroups using cross sectional study data from the China Adult Chronic Disease and Nutrition Surveillance in 2015 (CACDNS 2015). It also can provide scientific evidence to guide diet for the oldest-old and promote healthy aging in China.

## 2. Materials and Methods

### 2.1. Study Design and Samples

Data was obtained from the China Adult Chronic Disease and Nutrition Surveillance in 2015 (CACDNS 2015), which was carried out on stratified multistage random sampling design including 31 provinces, autonomous regions and municipalities [21]. The dwellers from these households were local residents or lived locally over 6 months. The study protocols were approved by the Ethical Committee of the Chinese Center for Disease Control and Prevention (No. 201519-B). All of the participants sighed the informed consent before participation.

A total of 2433 participants aged 80 and above had available data of food intake. The persons with energy intakes lower than 500 kcal/d or higher than 3700 kcal/d, the person who reported less than one day of dietary records or missing social-demographics data were excluded. Finally, 1929 subjects aged 80 and above were involved in this study.

### 2.2. Data Collection and Measurements

A standard set of questionnaires was designed to gather information on the individual social-demographics, diets and condiments. The dietary survey was carried out by well-trained dietary staff inquiring and recording the information via a face-to-face interview, including three-day consecutive 24-h dietary recalls at individual level, combing cooking oil and condiments weighing at household level (two weekdays and one weekend). The consumption of nutrient supplements or medicines were not analyzed in this study.

Subjects’ age, gender, region, education level, marital status and household income were collected by the individual social-demographic questionnaire. For older participants who were unable to provide the information by themselves, the questionnaire was answered by the person who took care of them or prepared the dishes for them.

### 2.3. Dietary Assessment

The condiments consumption from cooking oil and condiments weighing in household were divided to individual intake according to the number of meals at home and individual’s energy proportion among family members. The individual dietary consumption consists of food consumptions from 3-day 24-h dietary recalls and individual condiments consumption. Dietary energy and nutrients intakes were calculated based on Chinese Food Composition Tables [14,22].

The prevalence of deficiencies were evaluated using Chinese Dietary Reference Intakes (DRIs) [23]. The estimated average requirement (EAR) and estimated energy requirement (EER) were used to evaluate energy as well as nutrients intakes. The percentage of energy from carbohydrate and fat was used to compare with the acceptable macronutrient distribution ranges (AMDR) to assess whether their intakes were appropriate. For vitamin E, sodium and potassium, the daily intake was compared with the adequate intake (AI) since the EAR of these nutrients for the elderly aged 80 and above were not established in China yet. The total vitamin E intake was used to compare to AI, therefore the proportion of the subjects below AI may be underestimated.

### 2.4. Statistical Analysis

The post-stratification population sampling weights were applied to estimated nationally representative population levels for intake of nutrients, deriving from the sampling probability of the 2010 Chinese population census data aged 80 and above by regions, age and gender [16].

The weighted mean, standard error (SE), prevalence of deficiency of dietary energy and nutrients intakes were estimated by PROC SURVEYMEANS and PROC SURVEYFREQ. The complex sampling logistic regression analyze was used to analyze difference in the prevalence of deficient intakes between subgroups. All analyses were conducted with SAS software (v.9.4, SAS Institute Inc., Cary, NC, USA). A *p* < 0.05 was considered to be statistically significant.

## 3. Results

### 3.1. Participates Characteristics

A total of 1929 participants aged 80 and above were included in this study. The mean age was 83.3 years (SD 3.2), with 72.6% of 80–84 years and 27.4% of 85 years and above. 52.7% of participants were male and 54.2% were from rural areas. The general characteristics of the study population are presented in Table 1.

### 3.2. Assessment of Dietary Intake of Energy and Nutrients

The weighted mean and SE of daily dietary intakes of energy and nutrients and the prevalence of deficiency among the oldest-old were shown in Table 2. The dietary intakes of energy and most nutrients were all below the EAR or AI, except for fat, vitamin E, niacin, iron and sodium.

The dietary energy intake was 1436.0 kcal/d, and the dietary intakes of carbohydrate, protein and fat were 184.2 g/d, 43.9 g/d and 58.5 g/d, respectively. The prevalence of deficiency of energy and protein had overtaken 75%. The proportion of energy contribution from carbohydrate below 50% was around 40% in subjects. On the contrary, more than 60% of subjects in the percentage of energy from fat had over 30%. The daily dietary intakes of vitamin A, vitamin B_1_, vitamin B_2_, folate and calcium were extremely low among oldest-old, even did not meet the half of EAR, and the proportion of inadequacy were more than 90%. Furthermore, the prevalence of inadequacy of vitamin C, zinc, selenium and magnesium were also high, the proportion below the EAR was more than 60%. Approximately 30% of the subjects did not reach the AI for vitamin E, and more than 90% of subjects exceeded AI for sodium, while more than 90% did not reach AI for potassium. The mean intakes of niacin and iron have reached EAR, but approximately 15% were still faced with the risk of deficiency.

#### 3.2.1. Assessment of Dietary Intake of Energy and Nutrients between Urban and Rural Regions

Table 2 also showed the weighted mean and SE of daily dietary intakes of energy and nutrients and the prevalence of deficiency among the oldest-old between urban and rural regions. The daily dietary intakes of energy and all nutrients (except for sodium) were found to be slightly higher in urban elderly than rural. We also found that the dietary intakes of protein and potassium were the lowest, and the prevalence of inadequacies were the highest among all subgroups. The prevalence of inadequacy of protein, vitamin A, vitamin B_2_, niacin, iron, zinc and magnesium were significantly higher in rural elderly compared to urban elderly (*p* < 0.05). The proportion of the dietary vitamin E and potassium intakes below AI were higher in rural elderly than urban elderly (*p* < 0.05). There was no significant difference in the prevalence of inadequacy in other nutrients (*p* > 0.05).

#### 3.2.2. Assessment of Dietary Intake of Energy and Nutrients between Different Age Groups

The dietary intakes of energy and nutrients and the percentage of the oldest-old with inadequate intakes between age groups were shown in Table 3. The mean dietary intakes of energy and almost all nutrients were higher in 80–84 years group than in 85 years and above group, except for fat, vitamin E, vitamin C and selenium. Compared to 80–84 years group, the prevalence of inadequacy of energy, vitamin C, niacin, iron and magnesium were much higher in 85 years and above group (*p* < 0.05). Furthermore, the prevalence of deficiency of energy and vitamin B_1_ in the 85 years and above group were the highest, up to 80% and 96.7%. The reasonable proportion of carbohydrate intake was higher in 80–84 years group than 85 years and above (*p* < 0.05).

#### 3.2.3. Assessment of Dietary Intake of Energy and Nutrients between Two Genders

Table 4 showed the dietary intakes of energy and nutrients and the percentage of the oldest-old with inadequate intakes between two genders. Daily dietary intakes of energy and all nutrients were higher in male than female. We also found that the prevalence of deficiency of iron was highest in female elderly (20.4%). The prevalence of deficiency of zinc was highest in male elderly (82.2%), while was lowest in female elderly (48.4%). The prevalence of deficiency of energy, protein, vitamin B_1_, vitamin B_2_, niacin and zinc intakes were significantly higher in elderly men than elderly women (*p* < 0.05), but vitamin C, iron and magnesium were lower in elderly men than elderly women (*p* < 0.05). As for vitamin E and sodium, the percentage of subjects below AI was higher in female compared to male (*p* < 0.05).

#### 3.2.4. Assessment of Dietary Intake of Energy and Nutrients between Different Education Levels

The daily dietary intakes of energy and nearly all nutrients increased with education level, except for sodium. The dietary sodium intake was highest in the oldest-old graduated from primary school or middle school. The proportion of the subjects with inadequate intake of energy, protein, vitamin B_1_, vitamin B_2_, niacin, folate, calcium, iron, selenium and magnesium decreased with the education level (*p* < 0.05). However, the percentage of deficiency of zinc was highest in subjects with primary school or middle school and was lowest in high school and over (*p* < 0.05). As for vitamin E and potassium, the percentage of subjects below AI decreased with education level (*p* < 0.05). Table 5 shows details.

#### 3.2.5. Assessment of Dietary Intake of Energy and Nutrients between Different Material Status

The dietary intakes of energy and nutrients and the prevalence of inadequacy of the oldest-old were shown in Table 6. Compared to the oldest-old without spouse, the oldest-old with spouse consumed more energy and nutrients. There was no significant difference in the percentage of the subjects with inadequate energy and nutrients intakes between without spouse and with spouse after controlling other factors (*p* > 0.05), except for protein and potassium (compared to AI).

#### 3.2.6. Assessment of Dietary Intake of Energy and Nutrients between Different Household Income Levels

The mean dietary intakes of protein, vitamin A, vitamin B_2_, vitamin C, niacin, folate, calcium, iron, zinc, selenium and potassium increased as household income increased, but sodium decreased. The subjects from middle household income family consumed the lowest energy, carbohydrate, vitamin E and magnesium but the highest in fat. The prevalence of deficiency of protein, vitamin B_2_, folate and calcium declined as household income level increased (*p* < 0.05), but for magnesium was highest in middle-income household (*p* < 0.05). The prevalence of potassium below AI decreased as household income level increased (*p* < 0.05) (Table 7).

### 3.3. The Percentage of Energy Intake from Macronutrients

In this study, the percentage of energy from carbohydrate, protein and fat was 51.8%,12.3% and 36.1%, respectively. The percentage of energy from carbohydrate was higher in 80–84 years group compared to the 85 years and above group, in rural areas compared to urban areas, in male compared to female and in with spouse compared to without spouse. The percentage of energy from protein was higher in urban than in rural areas, and in with spouse than in without spouse. The proportion of energy from carbohydrate decreased as education level and household income level increased. The proportion of energy from protein increased as education level and household income level increased. Energy contribution from fat was higher in 85 years and above group compared to 80–84 years group, in urban areas compared to rural areas, in female compared to male, and in without spouse compared to with spouse. Energy contribution from fat was lower in the subjects with high school and above than others and was higher in subjects with middle household income than others. Table 8 showed the details.

## 4. Discussion

To our knowledge, this is the first study on the daily dietary intakes of energy and nutrients in Chinese elderly aged 80 and above on national level. Previous studies have analyzed the dietary daily nutrients intakes in some provinces or regions, but study on national level was limited. In this study, we deeply assessed the deficiency risk of the Chinese oldest-old and discovered the differences among age, gender, region, education level, marital status and household income level. We found that the majority of the elderly aged 80 and above in China had inadequate intake of energy and nutrients, especially in vitamin A, vitamin B_1_, vitamin B_2_, folate and calcium.

In the current study, we found that the average dietary energy intake of the oldest-old was 1436.0 kcal/d, the prevalence of deficiency for energy was 75.8% which was the same as old people aged 65 and above in 2015 [24]. Data from Newcastle 85+ Study showed that more than 80% of the cohort had lower energy intake than the EAR in very old people aged 85 years and above [25]. In addition, 78% of geriatric subjects did not consume the recommended daily intake for energy in India [26]. This suggested that the problem of insufficient energy intake among the oldest-old was widespread. After controlling other factors, we found that age, gender and education level were main factors affecting the deficiency rate of energy. Similar results were reported among Chinese adults [27]. The prevalence of inadequacy for energy increased with age and decreased with the increase of education level (*p* < 0.05). Nearly half of the oldest-old did not participate in physical activity and few participated in physical activity in the leisure time [28]. About four-fifths of the oldest-old suffered from at least one common chronic disease [17], and most of them lived with disease and cannot take care of themselves. Furthermore, nearly 90% were not well-educated and their incomes mainly came from child [19]. In current study, we clearly found that the prevalence of deficiency for almost all nutrients in the subjects graduated from high school or more were significantly lower than others (*p* < 0.05). Well-educated people have greater volition and ability to acquire health information which might help them regulate dietary behaviors, rather than following their instinctive appetite or preference [29]. Health education would probably be a useful tool to help them improve their dietary nutrition. However, the education level of the oldest-old in China was relatively low and more than half never attended school [19]. The dietary energy intake for male was higher than female, but EER satisfaction rate was significantly lower than female (*p* < 0.05). The higher dietary energy and nutrients requirements in males and different physical structures can explained partly. The dietary carbohydrate and protein intake were lower in the current study than that reported in CNNHS 2010–2012, but the dietary fat intake was slightly higher than 2010–2012 [10]. Most participants failed to meet the EAR for protein (76.5%), and merely 39.5% and 22.3% of subjects in the energy contribution from carbohydrate and fat reached the ADMR. While in Sweden, the proportion of protein and fat intake meeting Nordic nutrition recommendations (NNR) 2012 was more than 60%, but of carbohydrate intake was less than 30% among the elderly aged 70 years and over [30]. The old people are prone to muscle attenuation, and dietary protein intake is an effective way to delay it. The prevalence of inadequacy for protein was significantly different in all subgroups except for age group (*p* < 0.05). The difference in dietary protein intake between urban and rural areas was obvious, and the prevalence of deficiency in rural elderly was the highest among all subgroups. The 2010–2012 CHNNS reported that the prevalence of malnutrition of urban male and urban female aged 75 years and above were 7.6% and 6.7%, respectively, while rural male and female were 16.2% and 11.2% [11], which were consistent with the present results. This may be related to the level of urban economic development and the convenience and availability of food, indicating the need for specific public policies in different regions. Previous studies have showed that the marital status was association with dietary intakes and health condition [31,32], although the study sample was limited, our study also found lower mean protein intake in subjects without spouse with only 18.5% reaching the EAR. However, the number of the elderly who were widowed was increasing with age, the proportion of the oldest-old with spouse was merely 37.6% in China [18]. Further study on the relationship between marital status and dietary intakes in the elderly should be carried out. Household income level was the important factor to influence dietary intakes. In this study, high dietary protein intake and low prevalence of inadequacy were found in the subjects from high household income family. For the oldest-old, increasing the dietary intake of protein, especially high quality protein, can greatly improve the dietary quality and nutritional status. Although the prevalence of deficiency of energy and protein were so high, the prevalence of malnutrition was just 10.1% among the elderly aged 75 years and above [11]. According to DRIs [23], the EAR and RNI of protein in oldest-old are same as adults, and the EER of oldest-old was reckoned based on adults, therefore the recommendations may be overestimated. In the percentage of energy intake from macronutrients, the most serious question was too much consumption of fat. The energy contribution from fat in total population and all subgroups all exceeded the recommended proportion of 30% from the DRIs and close to or over 35%. More than 40% of the oldest-old consumed insufficient carbohydrate. It showed that the diet structure of the oldest-old was severely unreasonable. As for the elderly, the higher intake of fat was bound to increase the risk of chronic diseases such as dyslipidemia and hypertension [10]. Therefore, the elderly aged 80 and over should adjust their dietary pattern, increasing the intake of protein, decreasing the intake of fat and cultivating good eating habits.

Micronutrients deficiency was difficult to detect due to its inconspicuous performance, but will increase their risk of developing adverse health consequences among the geriatric population such as anemia, osteoporosis, joint pains, anorexia, weight loss, cognitive function decline, and malnutrition [33]. Micronutrient deficiency was common in the elderly aged 80 and above in China. In this study, the percentage of inadequate iron among the oldest old was more than as twice as that in Chinese elderly aged 75 and over living at home based on CNNHS 2010–2012 [33]. The deficiency of dietary iron was getting worse in the elderly aged 80 and above, especially living in rural, the elderly aged 85 and above, female, without spouse, ungraduated from primary school and from low household income level family, the prevalence was closed to or over 20%. What is more, the prevalence of deficiency for iron was approximately twice higher in female than male and in rural than in urban areas. This was largely due to the fact that the sources of dietary iron in the Chinese were mainly plant foods which absorption rate was not high. The dietary vitamin E intake in current study was similar to the residents aged 70 years and above in CHNS 2015 [13], and nearly 30% unreached the AI from DRIs. Although the consumption of potassium and sodium slightly declined from 2010–2012 to 2015 [11], the average dietary potassium intake has merely reached the half of AI, and sodium intake was three higher than AI and was twice higher than the recommendation of WHO. Several studies suggested that high sodium intake and higher sodium-potassium ratio were associated with significantly increased risk of stroke, CVD and all-cause mortality [34,35,36]. A prospective cohort study demonstrated that combined moderate sodium intake (3–5 g/day) with high potassium intake is associated with the lowest risk of mortality and cardiovascular events [36,37]. Reducing the consumption of salt has been the public concern in China, the oldest-old should also reduce dietary sodium intake to stable blood pressure. Under the situation of lower energy intakes in Chinese oldest-old, high nutrient densities of the diet are required to alleviate micronutrients deficiency. According to previous study, dietary supplement use can increased micronutrients intakes and reduced chronic diseases and total cancer risk, improved energy levels and enhanced mood [38,39]. Considering the decline of mastication and digestion in oldest old, using supplements may be the best approach to alleviate micronutrients deficiency.

There were several limitations in this study. Firstly, 3-day 24-h dietary recalls were applied to obtain food consumption, and the accuracy of dietary intakes was depended on the recall of individuals or their caregivers. While the visual and acoustic faculty were probably worse in participants, it would affect the estimation of dietary intakes. Secondly, not all foods were included in Chinese Food Composition Tables, and similar foods were used to replace them to estimate dietary intakes. What is more, some nutrients data was lacked in Chinese Food Composition Tables, which might underestimated the intake of dietary nutrients. Finally, nutrient supplements were not analyzed in this study, so the dietary intakes may be underestimated, such as folate.

## 5. Conclusions

The overall dietary intake was inadequate among the oldest-old in China and most existed in the risk of nutritional deficiency. Distinct disparities have existed in area, gender, education level and household income level. Insufficient energy and protein intakes, excess fat and sodium intakes and micronutrients deficiency were the major concern. The elderly living in rural areas, with lower education level and from lower household income level family were more vulnerable to nutrients deficiency. Further measures should be carried out to focus on key areas and population to improve the quantity and quality of dietary intake of the oldest-old in order to reduce the burden of health system and family.

## Figures and Tables

**Table 1:** Social-demographic distribution of participants aged 80 and above in CACDNS 2015.

	Total		Urban		Rural	
	N	%	N	%	N	%
Total	1929	100.0	884	45.8	1045	54.2
Age Group, year						
80–84	1400	72.6	631	71.4	769	73.6
≥85	529	27.4	253	28.6	276	26.4
Gender						
Male	1017	52.7	456	51.6	561	53.7
Female	912	47.3	428	48.4	484	46.3
Education Level *						
Ungraduated from primary school	1221	63.3	402	45.5	819	78.4
Primary school or middle school	508	26.3	293	33.1	215	20.6
High school and above	200	10.4	189	21.4	11	1.1
Marital Status **						
Without spouse	654	33.9	292	33.0	362	34.6
With spouse	1275	66.1	592	67.0	683	65.4
Household Income Level ***						
Low	525	27.2	100	11.3	425	40.7
Middle	514	26.7	253	28.6	261	25.0
High	526	27.3	416	47.1	110	10.5
Unclear	364	18.9	115	13.0	249	23.8

* Education Level: Ungraduated from primary school: ungraduated from primary school and illiteracy; High school and above: high school/technical secondary school/technical school, junior college, regular college course, graduate student and above. ** Marital Status: Without spouse: spinsterhood, widowed, divorce; With spouse: married, cohabitation, separation. *** Household Income Level: Low (<20,000 RMB), Middle (20,000–50,000 RMB), High (≥50,000 RMB) and Unclear (unknow or refuse to answer).

**Table 2:** Dietary daily intake of energy and nutrients and the prevalence of deficiency in the subjects aged 80 and above in urban and rural.

Nutrient	Total			Urban			Rural		
	Mean	SE	Below EAR (%)	Mean	SE	Below EAR (%)	Mean	SE	Below EAR (%)
Energy (kcal/d) ^#^	1436.0	16.5	75.8	1479.0	23.3	73.6	1401.6	19.9	77.6
Protein (g/d)	43.9	1.0	76.5	49.3	1.4	67.7 ^a^	39.7	0.8	83.5 ^a^
Fat (g/d) *	58.5	1.1	22.3 ^h^	60.7	1.5	22.3 ^h^	56.7	1.4	22.3 ^h^
Carbohydrate (g/d) *	184.2	3.0	39.5 ^g^	184.6	3.9	39.0 ^g^	183.9	3.8	40.0 ^g^
VitaminA (μgRAE/d) ^&^	221.4	9.4	93.2	263.7	14.0	90.5 ^a^	187.6	10.0	95.4 ^a^
VitaminE (mg/d) ^&^^	26.1	0.9	29.8	27.5	1.1	22.8 ^a^	25.0	1.3	35.4 ^a^
Vitamin B_1_ (mg/d)	0.6	0.0	94.4	0.6	0.0	93.5	0.6	0.0	95.1
Vitamin B_2_ (mg/d)	0.6	0.0	94.2	0.7	0.0	90.1 ^a^	0.5	0.0	97.5 ^a^
Vitamin C (mg/d)	65.6	2.3	76.1	74.4	3.9	71.9	58.6	2.0	79.3
Niacin (mgNE/d) ^&^	16.5	0.4	15.1	18.2	0.5	9.4 ^a^	15.2	0.4	19.7 ^a^
Folate (µg/d)	118.7	4.2	97.0	141.2	6.5	94.9	100.6	3.0	98.7
Calcium (mg/d)	300.0	11.5	97.9	369.5	18.3	96.2	244.4	6.9	99.2
Iron (mg/d)	15.1	0.3	15.8	16.3	0.4	10.7 ^a^	14.1	0.3	19.8 ^a^
Zinc (mg/d)	7.3	0.1	63.7	7.8	0.2	57.5 ^a^	6.9	0.1	68.6 ^a^
Selenium (μg/d)	30.2	1.0	88.6	34.7	1.5	84.7	26.5	0.9	91.7
Magnesium (mg/d)	198.5	4.0	81.4	220.8	5.5	74.8 ^a^	180.7	3.8	86.8 ^a^
Sodium (mg/d) ^	4086.6	96.9	6.2	3949.6	130.6	6.4	4196.4	128.9	6.0
Potassium (mg/d) ^	1132.1	30.3	92.3	1328.5	41.6	87.0 ^a^	974.8	22.9	96.6 ^a^

^#^: Compare to EER; ^: Compare to AI; *: fat: the percentage of energy from fat in 20–30%; carbohydrate: the percentage of energy from carbohydrate in 50–65%; ^&^: μgRAE/d: μg Retinol Activity Equivalents (RAE) per day; mgNE/d: mg Niacin Equivalents (NE) per day; ^a^: after controlling age, gender, marital status, education level and household income level, *p* < 0.05. ^h^: total: <20%: 11.1%, >30%: 66.6%; urban: <20%: 8.7%, >30%: 69.0%; rural: <20%: 13.0%, >30%: 64.7%. ^g^: total: <50%: 44.8%, >65%: 15.7%; urban: <50%: 49.4%, >65%: 11.6%; rural: <50%: 41.1%, >65%: 18.9%.

**Table 3:** Dietary daily intake of energy and nutrients and the prevalence of deficiency in the subjects aged 80 and above in different age groups.

Nutrient	80–84			≥85		
	Mean	SE	Below EAR (%)	Mean	SE	Below EAR (%)
Energy (kcal/d) ^#^	1460.1	19.2	73.9 ^a^	1384.0	21.5	80.0 ^a^
Protein (g/d)	44.5	1.0	75.5	42.6	1.2	78.5
Fat (g/d) *	57.9	1.2	23.9 ^h^	59.8	1.8	18.9 ^h^
Carbohydrate (g/d) *	190.9	3.3	40.5 ^ag^	169.7	3.6	37.3 ^ag^
Vitamin A (μgRAE/d) ^&^	229.0	10.5	92.6	205.1	11.3	94.7
Vitamin E (mg/d) ^&^^	26.1	0.9	29.7	26.2	1.4	30.2
Vitamin B_1_ (mg/d)	0.6	0.0	93.4	0.6	0.0	96.7
Vitamin B_2_ (mg/d)	0.6	0.0	93.6	0.5	0.0	95.5
Vitamin C (mg/d)	65.6	2.0	74.7 ^a^	65.8	4.9	79.0 ^a^
Niacin (mgNE/d) ^&^	16.8	0.4	13.2^a^	15.8	0.5	19.3 ^a^
Folate (µg/d)	120.3	4.1	97.1	115.0	5.3	96.7
Calcium (mg/d)	306.8	12.3	97.7	285.3	13.4	98.2
Iron (mg/d)	15.4	0.3	13.7 ^a^	14.4	0.4	20.2 ^a^
Zinc (mg/d)	7.4	0.1	62.6	7.1	0.2	66.0
Selenium (μg/d)	30.1	1.0	88.9	30.3	1.3	87.9
Magnesium (mg/d)	204.4	4.4	79.5 ^a^	185.7	4.7	85.6 ^a^
Sodium (mg/d) ^	4173.1	114.8	5.5	3899.5	144.6	7.6
Potassium (mg/d) ^	1162.0	31.7	91.6	1067.2	35.8	93.8

^#^: Compare to EER; ^: Compare to AI; *: fat: the percentage of energy from fat in 20–30%; carbohydrate: the percentage of energy from carbohydrate in 50–65%; ^&^: μgRAE/d: μg Retinol Activity Equivalents (RAE) per day; mgNE/d: mg Niacin Equivalents (NE) per day; ^a^: after controlling age, gender, marital status, education level and household income level, *p* < 0.05. ^h^: 80–84: <20%: 12.5%, >30%: 63.6%; ≥85: <20%: 8.0%, >30%: 73.0%. ^g^: 80–84: <50%: 41.7%, >65%: 17.8%; ≥85: <50%: 51.5%, >65%: 11.2%.

**Table 4:** Dietary daily intake of energy and nutrients and the prevalence of deficiency in the subjects aged 80 and above in different genders.

Nutrient	Male			Female		
	Mean	SE	Below EAR (%)	Mean	SE	Below EAR (%)
Energy (kcal/d) ^#^	1560.5	20.5	79.8 ^a^	1333.3	17.9	72.6 ^a^
Protein (g/d)	47.6	1.0	77.9 ^a^	40.9	1.1	75.3 ^a^
Fat (g/d) *	62.3	1.3	24.1 ^h^	55.3	1.2	20.9 ^h^
Carbohydrate (g/d) *	203.1	3.6	40.6 ^g^	168.6	3.2	38.6 ^g^
Vitamin A (μgRAE/d) ^&^	235.0	10.7	93.9	210.2	10.9	92.7
Vitamin E (mg/d) ^&^^	28.3	1.1	25.5 ^a^	24.3	1.0	33.4 ^a^
Vitamin B_1_ (mg/d)	0.6	0.0	95.5 ^a^	0.6	0.0	93.5 ^a^
Vitamin B_2_ (mg/d)	0.6	0.0	95.4 ^a^	0.5	0.0	93.2 ^a^
Vitamin C (mg/d)	69.1	2.7	71.7 ^a^	62.8	2.9	79.6 ^a^
Niacin (mgNE/d) ^&^	17.9	0.4	18.0 ^a^	15.3	0.4	12.8 ^a^
Folate (µg/d)	127.0	4.8	97.0	111.8	4.2	96.9
Calcium (mg/d)	326.1	11.5	97.8	278.5	12.6	97.9
Iron (mg/d)	16.3	0.3	10.1 ^a^	14.1	0.3	20.4 ^a^
Zinc (mg/d)	7.9	0.1	82.2 ^a^	6.8	0.1	48.4 ^a^
Selenium (μg/d)	33.0	1.1	85.7	27.9	1.1	90.9
Magnesium (mg/d)	215.2	4.5	76.6 ^a^	184.8	4.3	85.4 ^a^
Sodium (mg/d) ^	4614.1	129.8	4.2 ^a^	3651.5	98.7	7.8 ^a^
Potassium (mg/d) ^	1229.2	32.0	89.8	1051.9	32.3	94.4

^#^: Compare to EER; ^: Compare to AI; *: fat: the percentage of energy from fat in 20–30%; carbohydrate: the percentage of energy from carbohydrate in 50–65%; ^&^: μgRAE/d: μg Retinol Activity Equivalents (RAE) per day; mgNE/d: mg Niacin Equivalents (NE) per day; ^a^: after controlling age, gender, marital status, education level and household income level, *p* < 0.05. ^h^: male: <20%: 11.4%, >30%: 64.5%; female: <20%: 10.9%, >30%: 68.3%. ^g^: male: <50%: 42.8%, >65%: 16.6%; female: <50%: 46.5%, >65%: 14.9%.

**Table 5:** Dietary daily intake of energy and nutrients and the prevalence of deficiency in the subjects aged 80 and above in different education level.

Nutrient	Ungraduated from Primary School			Primary School or Middle School			High School and Above		
	Mean	SE	Below EAR (%)	Mean	SE	Below EAR (%)	Mean	SE	Below EAR (%)
Energy (kcal/d) ^#^	1382.3	18.5	77.1 ^a^	1495.5	27.9	76.4 ^a^	1656.7	40.9	65.5 ^a^
Protein (g/d)	40.1	0.8	81.4 ^a^	48.4	1.5	73.6 ^a^	59.0	1.8	49.4 ^a^
Fat (g/d) *	56.5	1.3	22.3 ^h^	60.9	1.8	21.9 ^h^	66.4	2.1	23.5 ^h^
Carbohydrate (g/d) *	179.2	3.4	39.2 ^g^	189.1	4.5	40.9 ^g^	206.7	7.1	38.5 ^g^
Vitamin A (μgRAE/d) ^&^	201.4	9.6	94.2	235.1	12.8	92.5	325.5	19.9	88.6
VitaminE (mg/d) ^&^^	25.1	1.1	34.1 ^a^	27.2	1.1	23.5 ^a^	30.3	2.5	16.4^a^
Vitamin B_1_ (mg/d)	0.6	0.0	95.6 ^a^	0.6	0.0	93.5 ^a^	0.8	0.0	88.7 ^a^
Vitamin B_2_ (mg/d)	0.5	0.0	96.6 ^a^	0.6	0.0	92.2 ^a^	0.8	0.0	82.8 ^a^
Vitamin C (mg/d)	59.1	1.9	78.6	76.6	6.2	74.4	83.0	4.0	62.3
Niacin (mgNE/d) ^&^	15.2	0.3	17.7 ^a^	18.0	0.6	13.0 ^a^	21.5	0.7	2.4 ^a^
Folate (µg/d)	105.1	3.1	98.4 ^a^	135.6	5.7	94.7 ^a^	169.3	10.6	92.7 ^a^
Calcium (mg/d)	257.2	7.3	98.9 ^a^	343.4	16.8	97.7 ^a^	486.0	29.8	90.9 ^a^
Iron (mg/d)	14.0	0.2	19.7 ^a^	16.4	0.5	11.0 ^a^	19.0	0.6	0.7 ^a^
Zinc (mg/d)	6.9	0.1	63.5 ^a^	7.8	0.2	66.8 ^a^	8.9	0.2	56.8 ^a^
Selenium (μg/d)	27.1	0.9	91.9 ^a^	34.1	1.5	85.7 ^a^	41.3	1.7	72.9 ^a^
Magnesium (mg/d)	181.4	3.3	86.2 ^a^	219.7	5.8	77.3 ^a^	263.0	9.4	58.5 ^a^
Sodium (mg/d) ^	4062.7	111.3	6.7	4203.5	178.8	5.1	3951.2	175.5	5.0
Potassium (mg/d) ^	1007.7	22.2	96.2 ^a^	1276.1	42.0	88.2 ^a^	1626.2	54.3	76.2 ^a^

^#^: Compare to EER; ^: Compare to AI; *: fat: the percentage of energy from fat in 20–30%; carbohydrate: the percentage of energy from carbohydrate in 50–65%; ^&^: μgRAE/d: μg Retinol Activity Equivalents (RAE) per day; mgNE/d: mg Niacin Equivalents (NE) per day; ^a^: after controlling age, gender, marital status, education level and household income level, *p* < 0.05. ^h^: ungraduated from primary school: <20%: 12.4%, >30%: 65.3%; Primary school or middle school: <20%: 9.3%, >30%: 68.7%; high school and above:<20%: 6.3%, >30%: 70.1%. ^g^: ungraduated from primary school: <50%: 42.9%, >65%: 17.9%; Primary school or middle school: <50%: 46.9%, >65%: 12.3%; high school and above: <50%: 52.6%, >65%: 8.9%.

**Table 6:** Dietary daily intake of energy and nutrients and the prevalence of deficiency in the subjects aged 80 and above in different marital status.

Nutrient	Without Spouse			With Spouse		
	Mean	SE	Below EAR (%)	Mean	SE	Below EAR (%)
Energy (kcal/d) ^#^	1353.2	20.6	76.6	1484.5	21.4	75.3
Protein (g/d)	40.6	1.1	81.5 ^a^	45.9	1.1	73.5 ^a^
Fat (g/d) *	56.8	1.4	19.4 ^h^	59.5	1.4	24.1 ^h^
Carbohydrate (g/d) *	170.6	3.5	39.4 ^g^	192.2	3.6	39.6 ^g^
Vitamin A (μgRAE/d) &	203.4	10.9	93.4	232.0	10.9	93.2
Vitamin E (mg/d) ^&^^	26.0	1.6	31.9	26.2	0.9	28.6
Vitamin B_1_ (mg/d)	0.6	0.0	95.1	0.6	0.0	94.0
Vitamin B_2_ (mg/d)	0.5	0.0	95.1	0.6	0.0	93.7
Vitamin C (mg/d)	62.4	3.7	79.1	67.5	2.6	74.3
Niacin (mgNE/d) ^&^	15.2	0.4	15.0	17.3	0.4	15.2
Folate (µg/d)	110.9	4.5	97.2	123.2	4.7	96.9
Calcium (mg/d)	271.0	11.8	98.6	317.0	13.5	97.5
Iron (mg/d)	14.2	0.3	19.5	15.6	0.3	13.6
Zinc (mg/d)	6.8	0.2	60.1	7.6	0.1	65.7
Selenium(μg/d)	27.9	1.2	91.0	31.5	1.1	87.2
Magnesium (mg/d)	184.0	4.6	86.7	207.0	4.6	78.4
Sodium (mg/d) ^	3955.4	142.3	6.0	4163.5	117.6	6.3
Potassium (mg/d) ^	1035.9	31.8	95.7 ^a^	1188.4	34.9	90.3 ^a^

^#^: Compare to EER; ^: Compare to AI; *: fat: the percentage of energy from fat in 20–30%; carbohydrate: the percentage of energy from carbohydrate in 50–65%; ^&^: μgRAE/d: μg Retinol Activity Equivalents (RAE) per day; mgNE/d: mg Niacin Equivalents (NE) per day; ^a^: after controlling age, gender, marital status, education level and household income level, *p* < 0.05. ^h^: without spouse: <20%: 9.9%, >30%: 70.8%; with spouse: <20%: 11.8%, >30%: 64.1%. ^g^: without spouse: <50%: 47.2%, >65%: 13.4%; with spouse: <50%: 43.4%, >65%: 17.0%.

**Table 7:** Dietary daily intake of energy and nutrients intakes and the prevalence of deficiency in the subjects aged 80 and above in different household income level.

Nutrient	Low			Middle			High			Unclear		
	Mean	SE	Below EAR (%)	Mean	SE	Below EAR (%)	Mean	SE	Below EAR (%)	Mean	SE	Below EAR (%)
Energy (kcal/d) ^#^	1431.3	32.6	76.9	1413.3	25.5	75.8	1487.4	29.8	74.0	1402.4	33.2	76.8
Protein (g/d)	40.0	1.1	83.3 ^a^	42.3	1.0	79.3 ^a^	51.2	1.7	63.4 ^a^	41.7	1.5	81.3
Fat (g/d) *	56.2	2.3	25.0 ^h^	59.9	2.1	19.7 ^h^	59.7	1.5	20.0 ^h^	58.1	2.1	25.6 ^h^
Carbohydrate (g/d) *	192.2	5.3	40.3 ^g^	177.0	4.0	40.5 ^g^	187.1	4.8	38.8 ^g^	179.0	5.6	37.9 ^g^
Vitamin A (μgRAE/d) ^&^	183.6	14.2	95.8	219.0	11.4	93.2	266.6	16.4	89.7	215.4	19.1	94.6
VitaminE (mg/d) ^&^^	27.2	1.5	30.3	25.6	1.2	30.3	26.2	1.0	22.6	25.1	2.6	38.8
Vitamin B_1_ (mg/d)	0.6	0.0	94.2	0.6	0.0	96.3	0.6	0.0	93.2	0.6	0.0	93.8
Vitamin B_2_ (mg/d)	0.5	0.0	98.0 ^a^	0.5	0.0	95.8 ^a^	0.7	0.0	87.1 ^a^	0.5	0.0	96.4
Vitamin C (mg/d)	57.1	2.2	80.7	68.4	5.6	76.9	73.4	3.0	69.0	63.0	3.2	78.2
Niacin (mgNE/d) ^&^	15.2	0.5	21.0	15.9	0.4	14.0	18.7	0.6	8.4	16.0	0.6	17.9
Folate (µg/d)	103.5	3.8	99.3 ^a^	111.6	4.0	98.1 ^a^	150.7	7.6	92.9 ^a^	105.1	5.4	97.8
Calcium (mg/d)	255.2	7.8	99.6 ^a^	272.2	9.3	99.1 ^a^	405.9	23.4	93.9 ^a^	254.2	12.1	99.4
Iron (mg/d)	14.5	0.4	19.0	14.6	0.3	16.2	16.8	0.6	9.3	14.2	0.4	19.7
Zinc (mg/d)	6.9	0.2	67.3	7.1	0.2	63.2	8.1	0.2	57.0	7.0	0.2	68.6
Selenium (μg/d)	25.6	1.0	92.5	29.6	1.2	89.8	36.1	1.9	82.1	29.1	1.4	90.4
Magnesium (mg/d)	190.7	5.5	82.9 ^a^	189.7	4.7	85.6 ^a^	227.2	6.9	71.1 ^a^	181.5	5.9	88.0
Sodium (mg/d) ^	4343.5	175.0	7.0	4158.7	169.2	5.5	3779.2	128.6	6.1	4048.6	176.5	6.2
Potassium (mg/d) ^	1002.6	26.8	96.2 ^a^	1087.5	30.6	95.5 ^a^	1389.3	52.4	83.2 ^a^	1016.9	38.4	95.2

^#^: Compare to EER; ^: Compare to AI; *: fat: the percentage of energy from fat in 20–30%; carbohydrate: the percentage of energy from carbohydrate in 50–65%; ^&^: μgRAE/d: μg Retinol Activity Equivalents (RAE) per day; mgNE/d: mg Niacin Equivalents (NE) per day; ^a^: after controlling age, gender, marital status, education level and household income level, *p* < 0.05. ^h^: low: <20%: 15.2%, >30%: 59.8%; middle: <20%: 11.1%, >30%: 69.2%; high: <20%: 8.2%, >30%: 71.8%; unclear: <20%: 9.3%, >30%: 65.1%. ^g^: low: <50%: 37.5%, >65%: 22.2%; middle: <50%: 44.8%, >65%: 14.7%; high: <50%: 50.1%, >65%: 11.1%; unclear: <50%: 47.9%, >65%: 14.2%.

**Table 8:** Daily dietary energy intake and the estimated percentage of energy intake from macronutrients in subjects.

	Energy Intake (kcal/d)		The Percentage of Energy from Macronutrients (%)					
			Carbohydrate		Protein		Fat	
	Mean	SE	Mean	SE	Mean	SE	Mean	SE
Total	1436.0	16.5	51.8	0.5	12.3	0.2	36.1	0.5
Age Group, year								
80–84	1460.1	19.2	52.8	0.6	12.3	0.2	35.2	0.5
≥85	1384.0	21.5	49.8	0.8	12.3	0.3	38.1	0.8
Region								
Urban	1479.0	23.3	50.5	0.7	13.4	0.3	36.3	0.6
Rural	1401.6	19.9	52.9	0.7	11.4	0.2	35.9	0.7
Gender								
Male	1560.5	20.5	52.5	0.6	12.3	0.2	35.4	0.6
Female	1333.3	17.9	51.3	0.6	12.3	0.2	36.7	0.6
Education Level								
Ungraduated from primary school	1382.3	18.5	52.3	0.6	11.7	0.2	36.1	0.6
Primary school or middle school	1495.5	27.9	51.2	0.8	13.0	0.3	36.1	0.7
High school and above	1656.7	40.9	49.8	0.9	14.5	0.3	35.9	0.9
Marital Status								
Without spouse	1353.2	20.6	50.9	0.6	12.0	0.2	37.4	0.6
With spouse	1484.5	21.4	52.4	0.6	12.5	0.2	35.4	0.6
Household Income								
Low	1431.3	32.6	54.2	0.9	11.3	0.2	34.8	0.9
Middle	1413.3	25.5	51.0	0.8	12.1	0.2	37.1	0.8
High	1487.4	29.8	50.5	0.7	13.7	0.3	36.0	0.6
Unclear	1402.4	33.2	51.5	0.9	12.0	0.3	36.7	0.9

## Data Availability

The data presented in this study are non-public.

## References

[1] United Nations, Department of Economic and Social Affairs, Population Division (2019). World Population Prospects 2019, Volume I: Compressor Tables.

[2] Tak Y.J., Lee J.G., Yi Y.H., Kim Y.J., Lee S., Cho B.M., Cho Y.H. (2018). Association of Handgrip Strength with Dietary Intake in the Korean Population: Findings Based on the Seventh Korea National Health and Nutrition Examination Survey (KNHANES VII-1), 2016. Nutrients.

[3] Kim E.-K., Kim H., Vijayakumar A., Kwon O., Chang N. (2017). Associations between fruit and vegetable, and antioxidant nutrient intake and age-related macular degeneration by smoking status in elderly Korean men. Nutr. J..

[4] Kim T.S., Chung J.W. (2019). Associations of Dietary Riboflavin, Niacin, and Retinol with Age-related Hearing Loss: An Analysis of Korean National Health and Nutrition Examination Survey Data. Nutrients.

[5] Bruins M.J., Van Dael P., Eggersdorfer M. (2019). The Role of Nutrients in Reducing the Risk for Noncommunicable Diseases during Aging. Nutrients.

[6] Mendonça N., Hill T.R., Granic A., Davies K., Collerton J., Mathers J.C., Siervo M., Wrieden W.L., Seal C.J., Kirkwood T.B.L. (2016). Micronutrient intake and food sources in the very old: Analysis of the Newcastle 85+ Study. Br. J. Nutr..

[7] Park H.J., Byun M.K., Kim H.J., Kim J.Y., Kim Y.I., Yoo K.-H., Chun E.M., Jung J.Y., Lee S.H., Ahn C.M. (2016). Dietary vitamin C intake protects against COPD: The Korea National Health and Nutrition Examination Survey in 2012. Int. J. Chron. Obstruct. Pulmon. Dis..

[8] Sun C., Wang R., Li Z., Zhang D. (2019). Dietary magnesium intake and risk of depression. J. Affect. Disord..

[9] Li Z., Wang W., Xin X., Song X., Zhang D. (2018). Association of total zinc, iron, copper and selenium intakes with depression in the US adults. J. Affect. Disord..

[10] Song P., Man Q., Li Y., Jia S., Fang Y., He L., Zhang J. (2019). Status of dietary energy and macronutrients intake among Chinese older adults from 2010 to 2012. J. Hyg. Res..

[11] Zhang J., Zhao L.Y. (2018). China National Nutrition and Health Surveillance Report: 2010–2013 Nutrition and Health Status of the Elderly in China.

[12] Wang L.S., Zhang B., Wang H.J., Du W.W., Zhang J.G., Wang Z.H. (2019). Intakes of energy and macronutrient among the elderly in nine provinces (autonomous region), China during 1991–2015. J. Hyg. Res..

[13] Wang L.S., Zhang B., Wang H.J., Du W.W., Zhang J.G., Wang Z.H. (2019). Secular trends in dietary micronutrient intakes among the elderly in nine provinces (autonomous regions) of China from 1991 to 2015. J. Environ. Occup. Med..

[14] Yu D.M., Zhao L.Y., Zhang J., Yang Z.Y., Yang L.C., Huang J., Fang H.Y., Guo Q.Y., Xu X.L., Ju L.H. (2021). China Nutrition and Health Surveys (1982−2017). CCDC Wkly..

[15] Ren Z.P., Xiong C., Zhou Z. (2019). China Fertility Report 2019. Dev. Res..

[16] National Bureau of Statistics of China (2012). Tabulation on the 2010 Population Census of the People’s Republic of China.

[17] Cui J., Mao F., Wang Z.H. (2016). Comorbidity of common chronic diseases among the elderly in China. Chin. J. Public Health.

[18] Tey N.P., Lai S.L., Teh J.K.L. (2016). The debilitating effects of chronic diseases among the oldest old in China. Maturitas.

[19] Dang J.W., Li J. (2019). Development Report on the Quality of Life for the Elderly in China (2019).

[20] Cao Z., Wang R., Cheng Y., Yang H., Li S., Sun L., Xu W., Wang Y. (2019). Adherence to a healthy lifestyle counteracts the negative effects of risk factors on all-cause mortality in the oldest-old. Aging (Albany N.Y.).

[21] Yang Y.X. (2005). Chinese Food Composition Table.

[22] Yang Y.X. (2009). Chinese Food Composition Table, 2nd ed.

[23] Chinese Nutrition Society (2014). Chinese Dietary Reference Intakes (2013).

[24] Zhao F.L., Fang H.Y., Zhao L.Y., Mu D., Guo Q.Y., Ju L.H., He L. (2021). Intakes of dietary energy and macronutrients among the elderly aged 65 and above in China in 2015. J. Hyg. Res..

[25] Mendonça N., Hill T.R., Granic A., Davies K., Collerton J., Mathers J.C., Siervo M., Wrieden W.L., Seal C.J., Kirkwood T.B.L. (2016). Macronutrient intake and food sources in the very old: Analysis of the Newcastle 85+ Study. Br. J. Nutr..

[26] Gupta A., Khenduja P., Pandey R.M., Sati H.C., Sofi N.Y., Kapil U. (2017). Dietary Intake of Minerals, Vitamins, and Trace Elements Among Geriatric Population in India. Biol. Trace Elem. Res..

[27] Jiang H., Zhang J., Du W., Su C., Zhang B., Wang H. (2021). Energy intake and energy contributions of macronutrients and major food sources among Chinese adults: CHNS 2015 and CNTCS 2015. Eur. J. Clin. Nutr..

[28] Oiyang Y.F., Wang H.J., Wang Z.H., Song Y.Q., Zhang B. (2019). Physical activity among elderly residents in 15 provinces of China in 2015. J. Environ. Occup. Med..

[29] Zhu Z., Yang X., Fang Y., Zhang J., Yang Z., Wang Z., Liu A., He L., Sun J., Lian Y. (2020). Trends and Disparities of Energy Intake and Macronutrient Composition in China: A Series of National Surveys, 1982–2012. Nutrients.

[30] Samuelsson J., Rothenberg E., Lissner L., Eiben G., Zettergren A., Skoog I. (2019). Time trends in nutrient intake and dietary patterns among five birth cohorts of 70-year-olds examined 1971–2016: Results from the Gothenburg H70 birth cohort studies, Sweden. Nutr. J..

[31] Vinther J.L., Conklin A.I., Wareham N.J., Monsivais P. (2016). Marital transitions and associated changes in fruit and vegetable intake: Findings from the population-based prospective EPIC-Norfolk cohort, UK. Soc. Sci. Med..

[32] Lee S., Cho E., Grodstein F., Kawachi I., Hu F.B., A Colditz G. (2005). Effects of marital transitions on changes in dietary and other health behaviours in US women. Int. J. Epidemiol..

[33] Liu Z., Zhao L., Man Q., Wang J., Zhao W., Zhang J. (2019). Dietary Micronutrients Intake Status among Chinese Elderly People Living at Home: Data from CNNHS 2010–2012. Nutrients.

[34] O’Donnell M., Mente A., Rangarajan S., McQueen M.J., O’Leary N., Yin L., Liu X., Swaminathan S., Khatib R., Rosengren A. (2019). Joint association of urinary sodium and potassium excretion with cardiovascular events and mortality: Prospective cohort study. BMJ.

[35] Cook R.N., Appel L.J., Whelton P.K. (2016). Sodium Intake and All-Cause Mortality over 20 Years in the Trials of Hypertension Prevention. J. Am. Coll. Cardiol..

[36] Yang Q., Liu T., Kuklina E.V., Flanders W.D., Hong Y., Gillespie C., Chang M.-H., Gwinn M., Dowling N., Khoury M.J. (2011). Sodium and potassium intake and mortality among US adults: Prospective data from the Third National Health and Nutrition Examination Survey. Arch. Intern. Med..

[37] Jayedi A., Ghomashi F., Zargar M.S., Shab-Bidar S. (2019). Dietary sodium, sodium-to-potassium ratio, and risk of stroke: A systematic review and nonlinear dose-response meta-analysis. Clin. Nutr..

[38] Ruxton C.H., Derbyshire E., Toribio-Mateas M. (2016). Role of fatty acids and micronutrients in healthy ageing: A systematic review of randomised controlled trials set in the context of European dietary surveys of older adults. J. Hum. Nutr. Diet.

[39] Blumberg J.B., Frei B., Fulgoni V.L., Weaver C.M., Zeisel S.H. (2017). Contribution of Dietary Supplements to Nutritional Adequacy in Various Adult Age Groups. Nutrients.

